# Reviewing the peer review

**DOI:** 10.4103/0253-7613.40480

**Published:** 2008

**Authors:** Chetna Desai

**Affiliations:** Assistant Editor, Indian Journal of Pharmacology, Department of Pharmacology, B.J. Medical College, Ahmedabad - 380 016, Gujarat, India. E-mail: chetna99@gmail.com

Peer review or refereeing is a process to subject a scholarly work, research or an idea to the scrutiny of others who are experts in the same field. The development of this process has been interesting. Different editors have employed varying styles of peer review in the past. The *Lancet*, pre-1976, did not implement peer review, as they considered it unimportant. Some journals, such as the *Journal of the American Medical Association* (*JAMA*), usually employed an internal review only and, sometimes the outside experts[1]. The *British Medical Journal*, however, sent every non-editorial submission to a recognized expert by at least 1893[2]. By the late 20^th^ century, peer review had been adopted by most biomedical journals.

Today, peer review is an integral step in the publication of a scientific literature. Much is at stake on this particular step, be it the acceptance or otherwise of an article or the quality of the journal itself. It is not surprising therefore that journals, editors and editorial boards lay a great emphasis on the selection of quality peer reviewers. The process lends a semblance of respectability and legitimacy to the article and the journal as well. It ensures certain minimum standards of publication and prevents dissemination of false claims and unacceptable claims.

The moot question, however, is whether it really works? And if yes, how to make it work best? The first question may sound preposterous to the believers of this sacrosanct process, but it is not that the question has been raised for the first time. Traditionally, it is an accepted fact that peer review helps weed out bad papers and bad research and also improves upon better ones. The process not only educates the author, but the manuscript assessors and editors too! Thus, how do we make this process work best? There has been quite a bit of brainstorming on this issue among the members of the editorial board of this journal. We wish to share a few of them with you.

Once you get an invitation to review, it helps the editorial board to receive a note of acceptance to review or otherwise from you. The decision to review or not will be difficult. This journal usually sends manuscripts based on the expertise of the reviewer (as recorded in our electronic database). However, in case of any constraints, do inform us. The review process is one of the multiple steps to the final publication/rejection; an inordinate delay at this stage affects the time schedule of the article cycle.

Changed addresses and obsolete email ids of the reviewers are another bottleneck. This journal is making efforts to overcome this by updating the address and other particulars of the reviewers. Existing as well as prospective reviewers may refer to the announcement made about it in an earlier issue (October 2007) of this journal. Do send in all the relevant information, so that we may strengthen our database.

There is nothing like a perfect review. Each reviewer has a distinct style of reviewing. However, guidelines help maintain a semblance of uniformity and simplify the job of the editors, especially when there are conflicting opinions. We have developed abbreviated guidelines for reviewing a manuscript for *IJP*, for perusal by the referees. In addition, a scoring system that helps assess the manuscripts objectively has been developed. We hope that our reviewers make the best use of these. The editor primarily needs to know whether the paper is suitable for publication or not; however, a good review also informs the author and the editor about the limitations of the paper and how these can be overcome. Manuscripts, especially the initial few, are precious to an author. They are prepared after endless drafts, checks and counterchecks and umpteen graphs, figures, photographs and signatures. More often than not, the author looks forward eagerly to the responses, especially the younger ones and the opinion of the reviewer. While it is a privilege to be a reviewer, it is also the responsibility of the manuscript assessor to do adequate justice to it. That of course does not mean that the reviewer goes into an “accept” mode but it rather means that a studied decision/opinion is given after thorough research, thought and analysis. An opinion can be made more meaningful and criticism more constructive for the author as he/she could utilize these for revision or resubmission in the same or other journal, in case of rejection. Usually, only technical or scientific opinion about the manuscript are sought from the reviewer; however, linguistic corrections to improve the quality without compromising the underlying message is welcome and an added bonus to the editors and the authors too!

Research suggests that the best peer reviewers are aged under 40 years, trained in epidemiology or statistics and live in North America![3] Training is said to have a variable role on the quality of reviews. But we believe that the quality of review depends on how much time, effort and expertise a reviewer is willing to invest and this journal certainly looks forward to such valuable investments. We currently have a varied and experienced team of senior reviewers who are the backbone of our peer-review process. However, we also wish to build a team of young reviewers, who with a little bit of training/experience could foray into this academically challenging task. This would go a long way in building a sound panel, which will hold good for years to come.

Online review process is increasingly being adopted by journals to expedite the process. However, it does meet with some reservations from the reviewers who do not have continuous access to the electronic media or those who are not familiar/comfortable with the process. While electronic “comforts” obviously cannot compensate for good reviewers, it is a long-term endeavor of this journal to eventually adopt this process fully. This needs the enthusiasm and acceptance of our esteemed reviewers.

Reviewing is generally a thankless job in terms of monetary returns. Few journals pay for the reviewers. The hidden reward is the contribution made to science and research, but it needs idealism, time and effort! Let us make an effort to strengthen this process, for the benefit of this journal.
